# GPT-4o’s competency in answering the simulated written European Board of Interventional Radiology exam compared to a medical student and experts in Germany and its ability to generate exam items on interventional radiology: a descriptive study

**DOI:** 10.3352/jeehp.2024.21.21

**Published:** 2024-08-20

**Authors:** Sebastian Ebel, Constantin Ehrengut, Timm Denecke, Holger Gößmann, Anne Bettina Beeskow

**Affiliations:** Department of Diagnostic and Interventional Radiology, University of Leipzig, Leipzig, Germany; Hallym University, Korea

**Keywords:** Artificial intelligence, Consultants, Europe, Interventional radiology, Medical students

## Abstract

**Purpose:**

This study aimed to determine whether ChatGPT-4o, a generative artificial intelligence (AI) platform, was able to pass a simulated written European Board of Interventional Radiology (EBIR) exam and whether GPT-4o can be used to train medical students and interventional radiologists of different levels of expertise by generating exam items on interventional radiology.

**Methods:**

GPT-4o was asked to answer 370 simulated exam items of the Cardiovascular and Interventional Radiology Society of Europe (CIRSE) for EBIR preparation (CIRSE Prep). Subsequently, GPT-4o was requested to generate exam items on interventional radiology topics at levels of difficulty suitable for medical students and the EBIR exam. Those generated items were answered by 4 participants, including a medical student, a resident, a consultant, and an EBIR holder. The correctly answered items were counted. One investigator checked the answers and items generated by GPT-4o for correctness and relevance. This work was done from April to July 2024.

**Results:**

GPT-4o correctly answered 248 of the 370 CIRSE Prep items (67.0%). For 50 CIRSE Prep items, the medical student answered 46.0%, the resident 42.0%, the consultant 50.0%, and the EBIR holder 74.0% correctly. All participants answered 82.0% to 92.0% of the 50 GPT-4o generated items at the student level correctly. For the 50 GPT-4o items at the EBIR level, the medical student answered 32.0%, the resident 44.0%, the consultant 48.0%, and the EBIR holder 66.0% correctly. All participants could pass the GPT-4o-generated items for the student level; while the EBIR holder could pass the GPT-4o-generated items for the EBIR level. Two items (0.3%) out of 150 generated by the GPT-4o were assessed as implausible.

**Conclusion:**

GPT-4o could pass the simulated written EBIR exam and create exam items of varying difficulty to train medical students and interventional radiologists.

## Graphical abstract

[Fig f1-jeehp-21-21]

## Introduction

### Background/rationale

Chat generative pretrained transformer (ChatGPT) is an online generative artificial intelligence (AI) platform based on a large language model (LLM), produced by OpenAI [1]. Since its release in November 2022, ChatGPT has been revised several times, and its most advanced version is currently commercially available as GPT-4o.

Interventional radiology is a growing field with an increasing number of procedures [2]. This is offset by a shortage of interventional radiologists in many regions, which means that there is a need to train a sufficient number of high-quality junior radiologists [3]. Therefore, the Cardiovascular Interventional Radiology Society of Europe (CIRSE) has created a curriculum for interventional radiology that covers all relevant topics. In addition, CIRSE has created the European Board of interventional Radiology (EBIR) exam, which tests all subject areas that an interventional radiologist must master. In the written part of the EBIR exam, candidates have to answer items on various areas of interventional radiology [4]. The EBIR certificate therefore serves as an instrument for the structured training of interventional radiologists and also indicates that the holders can perform procedures with a high level of patient safety and quality. Online platforms such as the CIRSE Academy or CIRSE Library, in which simulated exam items are available, can be used to prepare for the EBIR exam. Nevertheless, the EBIR exam is very comprehensive and requires detailed knowledge in the field of interventional radiology to pass, which in turn necessitates adequate training in advance and thorough preparation.

### Objectives

This study aimed to examine whether GPT-4o can answer these simulated exam items correctly and to determine whether it can be used to create exam items for medical students, young doctors, and EBIR candidates to prepare for the EBIR exam.

## Methods

### Ethics statement

Ethical approval was not necessary as the study did not use any personal data.

### Study design

This was a descriptive study investigating the performance of GPT-4o on the EBIR exam and possible applications for training interventional radiologists. It was described according to the STROBE (Strengthening the Reporting of Observational Studies in Epidemiology) statement available at https://www.strobe-statement.org/.

### Setting

We created 3 sets of items. For the first set (CIRSE for EBIR preparation [CIRSE Prep], n=370), we took the EBIR preparation items from each chapter of the 37 topics listed in Supplement 1 from the CIRSE academy.

For the second and third exam setsfor medical students (n=370) and EBIR candidates (n=370), respectivelywe asked GPT-4o to generate 10 multiple-choice items on each of the 37 topics, at the difficulty level for medical students and for EBIR candidates, as shown in Supplement 2.

The GPT-4o generated items for the student level and EBIR level were generated with the following commands: “Please create 5 multiple-choice items (answer options ad) on the topic XYZ (see Table 1) at the difficulty level of the EBIR exam and indicate the correct answers,” or “Please create 5 multiple-choice items (answer options ad) on the topic XYZ (Table 1) with a difficulty level for medical students and indicate the correct answers.” The output of the items was as follows: “Certainly! Here are 5 advanced-level multiple-choice items on the topic XYZ for a difficulty level comparable to the EBIR exam:” or “Sure, here are 5 multiple-choice items on the topic XYZ suitable for medical students:”

The 3 exam sets are available in Supplement 3. For all items, we asked GPT-4o to randomly select 50 items from each set, including items from each topic area. Finally, 150 items from the 3 exam sets were selected. This work was done from April to July 2024.

### Participants

These items were given to 4 participants in a blinded fashion: an EBIR holder who took the EBIR exam in 2020, a consultant who has completed training in interventional radiology, a resident in the middle of interventional radiology training, and a medical student before the final exam. The participants took part spontaneously, with no time to prepare, and did not know that the items were taken from CIRSE or were created by GPT-4o and were asked to answer all items in an exam-like manner by themselves without any external help.

### Variables

The primary outcome was the score of GPT-4o and 4 participants for examinations.

### Data sources/measurements

All 370 CIRSE Prep items were given to GPT-4o via copy-paste without any further instructions sequentially in one chat session per topic area. The answers given by GPT-4o were saved as a text file and compared to the answers given by CIRSE by one reader. To determine the retest variability, all incorrectly answered items were given to GPT-4o again 1 week after the first exposure in the same manner as before. The incorrect answers were then analyzed with regard to their error quality, whereby a distinction was made between statistical errors (example: the disease was correctly identified but the prevalence was incorrectly classified) and logical errors (example: GPT-4o recognizes that angiography has a more favorable risk profile than surgery with the same chances of recovery but names surgery as the best treatment method). The prompts for the exam items and the way in which GPT-4o answered the items are illustrated in Supplement 2.

All responses provided by GPT-4o and the human participants were checked by one reader (S.E., EBIR and EBIR Endovascular Specialist holder) for correctness and plausibility. For each correct answer, 1 point was given. A total score of at least 25 points per set was considered a pass. For GPT-4o’s responses to the CIRSE Prep items, a total score of at least 50% of the points (185 points) was counted as a pass. For each module, a minimum score of 5 points was counted as a pass, but this was not relevant for passing the overall examination.

### Bias

Four participants were recruited on a voluntary basis; thus, we may not be able to accurately evaluate the competence of the group.

### Study size

No sample size estimation was required, since this was a descriptive study.

### Statistical methods

Descriptive statistics were performed using IBM SPSS Statistical Software ver. 28.0 (IBM Corp.). All variables were given as mean±standard deviation.

## Results

### GPT-4o’s competency in answering the 370 CIRSE Prep items

GPT-4o could answer 248 of the 370 CIRSE Prep items (67.0%) correctly, thus passing the exam. The score in the individual subject areas ranged from 5 to 10 and averaged 7.5±1.6, meaning that each individual subject area was assessed as a pass. There were 3 different syntaxes in which GPT-4o answered the items (Supplement 2). Re-entering the incorrectly answered items gave the same results; the items were answered incorrectly again. No connection to subject areas could be found within the incorrectly answered items. There was a statistical error in 41 items and a logical error in 51 items out of 122 incorrect answers.

### Human answers to the 3 exam sets

The results of the human participants answering 3 exam sets, each containing 50 items, can be seen in Table 1.

### Plausibility of the items created by GPT-4o

Two (0.3%) of 740 itemsgenerated by GPT-4o were assessed as implausible each one for student level and board level. For 1 item, more than 1 option was correct for student level. Additionally, 1 incorrect answer option was given as correct in 1 item for board level.

## Discussion

### Key results

This present study showed that GPT-4o could answer the CIRSE Prep items, which are otherwise very difficult and can only be answered with certainty after intensive study of the field of interventional radiology. It was also shown that GPT-4o can be used to generate exam items of varying difficulty for interventional radiology.

### Interpretation

The varying levels of difficulty evident in the items generated by GPT-4o (student-level and EBIR) are clearly demonstrated by the disparate results achieved by participants with varying levels of expertise. All participants were successful in passing the examination at the medical student level. The medical student with the lowest level of expertise in interventional radiology was unable to pass the GPT-4o-generated EBIR exam, as was the resident with a slightly higher level of expertise in interventional radiology, although he was able to achieve a higher score in the GPT-4o-generated EBIR exam. The consultant with interventional radiology training also failed, but scored higher than the resident. The EBIR holder with the highest interventional radiology expertise was able to pass the exam. The authors therefore conclude that GPT-4o can be used not only in the context of EBIR examinations, but also to generate end-of-year examinations with an increasing level of difficulty over the years. This could be used to test residents or young consultants during their interventional radiology training.

In addition to passing the written EBIR examination, proof of a certain number of interventional radiology procedures carried out and proof of specialist certification in radiology are required to obtain the certificate. Although it was shown that the generative AI platform could pass the written EBIR exam, the other requirements for obtaining the certificate can only be met by a human. In addition, the fact that GPT-4o only achieved 67.0% accuracy and not 100% shows that pure factual knowledge is obviously not enough. The authors believe that clinical experience and the ability to draw conclusions and perform deductions as only a human can is more important.

Societies such as CIRSE and national radiological societies provide various online platforms where radiological and interventional radiological knowledge is available in text form. In addition, a large number of scientific articles on interventional radiology are freely available as open access, so it can be assumed that these data were also used to train GPT-4o. This explains why GPT-4o was able to answer most of the CIRSE EBIR preparation items correctly and to create items on interventional radiology at different levels of difficulty.

This study showed that the examination questions created by the LLM can be used to test medical students, residents, specialists, and EBIR candidates. Although only 0.3% of the LLM questions were rated as implausible, it is always necessary to check the plausibility of the questions and answers generated by the generative AI platforms and not accept them as true without reflection.

### Comparison with previous studies

The ability of GPT-4o to pass medical exams has already been demonstrated in a shoulder and elbow exam and in an orthopedic exam [5,6]. In both studies, the generated AI platforms were able to achieve the required minimum score in the respective exams and the answers could be logically justified in each case, which is consistent with the results of the present study. Our study complements aspects of training and learning. The generative AI platform was not only used to solve existing exams, but also to create new exam items with which human participants can be trained.

Other working groups have also used generative AI platforms including GPT-4o to answer the EBIR pre-assessment items of the CIRSE, showing that 67% of the items could be answered correctly, which corresponds exactly to the results of our study [7]. In our view, however, the benefit of generative AI platforms in the context of medical exams is not to let AI solve the exam, but to use AI to train and support people or medical students to learn and thus pass the exams, which is why our study focused on evaluating exam items generated by the generative AI platform.

In a study by Stengel et al. [8], the ethical issues surrounding the use of AI in the context of medical treatments were discussed using the example of a neurosurgical exam. It was discussed that if an AI can pass an exam, no human or natural intelligence is apparently necessary for this, implying the possibility of cheating on medical exams by using AI without permission. As a possible solution, it was proposed that oral exams could solve this dilemma, as it is not possible to use AI there without being noticed.

The EBIR exam was designed by CIRSE to test interventional radiologists on clinical knowledge, diagnostic skills, interventional (technical) skills, safety, and procedural effectiveness [2,4]. The EBIR certificate is intended to indicate a high level of expertise and therefore high quality in the field of interventional radiology. In some countries, such as Germany, there is no separate sub-specialization for interventional radiology, so the EBIR certificate is equivalent to a kind of specialist title for interventional radiology and represents the highest possible qualification in the field [3]. Given the shortage of young talent and the ever-increasing demand, it is essential to ensure continuous, high-quality training of young colleagues in interventional radiology.

### Limitations

GPT-4o was not tested on real EBIR exam items, but only on the CIRSE preparation items, because the actual EBIR exam items are not available online. However, the aim of this study was not to evaluate the actual performance of GPT-4o in the EBIR exam, but to show the potential advantages and possible applications of GPT-4o in the field of interventional radiology exams and training.

### Conclusion

The above results showed that GPT-4o is capable of passing a simulated written EBIR exam, and generative AI platforms can be used to create meaningful interventional radiology exam items for training young colleagues in interventional radiology.

## Figures and Tables

**Figure f1-jeehp-21-21:**
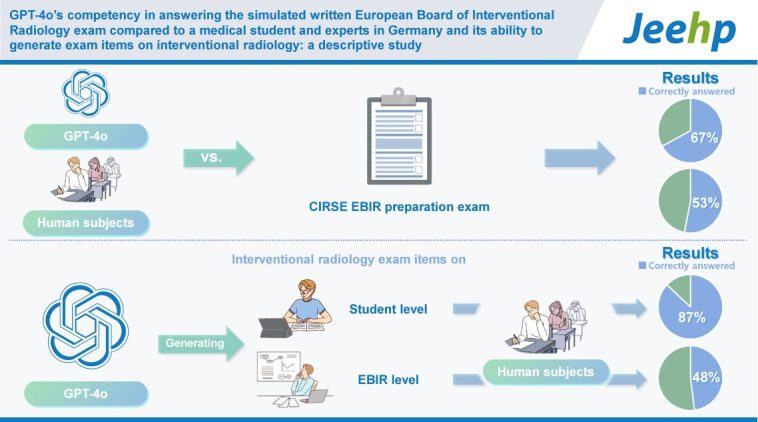


**Table 1.** Scores of the participants when answering the CIRSE for EBIR preparation items and 2 item sets generated by GPT-4o, including 50 items in each set

	No. of correct answer (%)		
	CIRSE set	Student level set	EBIR level set
EBIR holder	37 (74.0)	44 (88.0)	33 (66.0)
Consultant	25 (50.0)	46 (92.0)	24 (48.0)
Resident	21 (42.0)	41 (82.0)	22 (44.0)
Medical student	23 (46.0)	42 (84.0)	16 (32.0)

CIRSE, Cardiovascular and Interventional Radiology Society of Europe; EBIR, European Board of Interventional Radiology.

## Supplementary materials

Supplementary files are available from Harvard Dataverse: https://doi.org/10.7910/DVN/KMH2UC

Supplement 1. Thirty-seven topics of the Cardiovascular and Interventional Radiology Society of Europe academy.

Supplement 2. Four main prompts for GPT-4o used in this study.

Supplement 3. Fifty European Board of Interventional Radiology preparation items randomly selected from 370 items for 37 topics of the Cardiovascular and Interventional Radiology Society of Europe academy.

Supplement 4. Audio recording of the abstract.
