# Supplementary material for: GPT-4o’s competency in answering the simulated written European Board of Interventional Radiology exam compared to a medical student and experts in Germany and its ability to generate exam items on interventional radiology: a descriptive study
Source: J Educ Eval Health Prof. 2024 Aug 20;21:21. doi: 10.3352/jeehp.2024.21.21 (PMC11894030; doi:10.3352/jeehp.2024.21.21)
Supplement: Supplementary file 2 — Supplement 2. Four main prompts for GPT-4o used in this study. [file jeehp-21-21-suppl2.docx]

Supplement 2:

Please create 5 multiple choice questions (answer options a-d) on the topic of colorectal liver metastases at the difficulty level for experienced experts in interventional radiology (EBIR). And indicate the correct answers.

ChatGPT

Certainly! Here are five multiple-choice questions on the topic of colorectal liver metastases (CRLM), designed for experienced experts in interventional radiology (European Board of Interventional Radiology - EBIR) level:

Please create 5 multiple choice questions (answer options a-d) on the topic of colorectal liver metastases at the difficulty level for medical students. And indicate the correct answers.

ChatGPT

Certainly! Here are five multiple-choice questions on the topic of colorectal liver metastases (CRLM), tailored for medical students:

The rationale behind the efficacy of thermal ablation for treating liver tumours is the absolute sensitivity of any biological tissue to the heat. According to this concept, which is the range of temperature for achieving a “near instantaneous coagulative necrosis”? Select one:

a. 60°C – 100°C

b. 46°C - 48°C

c. 50°C – 52°C

d. 110°C – 120°C

ChatGPT

The range of temperature required to achieve "near instantaneous coagulative necrosis" in the treatment of liver tumors through thermal ablation is 60°C – 100°C. At these temperatures, the heat causes rapid and irreversible damage to cellular proteins and cell membranes, leading to immediate cell death. This range is optimal for effectively destroying tumor cells while minimizing damage to surrounding healthy tissue. The other temperature ranges mentioned are either too low to cause instantaneous necrosis or too high and thus potentially harmful to surrounding tissues.

A 56-year-old male was referred for TIPS. His liver was evaluated by CT angiography, depicting severe liver atrophy, a partial extrahepatic portal vein bifurcation and large volume ascites. Which is the most serious potential procedural complication of performing TIPS in this patient? Select one: a. Liver capsule perforation b. Extrahepatic portal vein laceration and massive intraperitoneal bleeding c. Inadvertent puncture of the bile duct d. Puncture of the hepatic artery

ChatGPT

In evaluating the potential procedural complications of performing a Transjugular Intrahepatic Portosystemic Shunt (TIPS) in a 56-year-old male with severe liver atrophy, a partial extrahepatic portal vein bifurcation, and large volume ascites, it's important to consider the specific anatomical challenges and risks associated with these conditions. Let's analyze each option:

1. **Liver Capsule Perforation**: Given the severe liver atrophy, there is less parenchymal tissue to work with, which could increase the risk of perforating the liver capsule during the TIPS procedure. However, while this is a concern, it might not be the most serious complication compared to others listed.
2. **Extrahepatic Portal Vein Laceration and Massive Intraperitoneal Bleeding**: The presence of a partial extrahepatic portal vein bifurcation poses a significant risk during the TIPS procedure. An incorrect needle pass could lead to laceration of the extrahepatic portion of the portal vein, which could result in massive intraperitoneal bleeding. This complication is potentially life-threatening and could be considered the most serious risk in this context.
3. **Inadvertent Puncture of the Bile Duct**: While the inadvertent puncture of the bile duct is a known complication of the TIPS procedure, its likelihood and severity might be less compared to the risk of extrahepatic portal vein laceration, especially in the context of the patient's specific anatomical challenges.
4. **Puncture of the Hepatic Artery**: Accidental puncture of the hepatic artery is a possible complication during TIPS. However, given the specific details of this patient's case (severe liver atrophy and a partial extrahepatic portal vein bifurcation), this might not be the most significant risk compared to the possibility of portal vein laceration.

Based on the patient’s specific anatomical considerations, the most serious potential procedural complication in this case would be **b. Extrahepatic portal vein laceration and massive intraperitoneal bleeding**. This risk is accentuated due to the presence of a partial extrahepatic portal vein bifurcation, which can complicate the procedure and increase the likelihood of severe bleeding. It is essential that such procedures be carried out by highly skilled and experienced interventional radiologists, and the patient's unique anatomical challenges should be thoroughly evaluated and planned for before proceeding with TIPS.

A 45-year-old female with blunt abdominal trauma shows free abdominal fluid on FAST-sonography with a falling haematocrit. What is the most appropriate management? Select one: a. Stabilise the patient. Then transfer to CT immediately. Depending on the findings consider Angio-suite or operating theater b. Transfer to the operating theater because of hemodynamic instability c. Transfer the patient immediately to the Angio-suite because of suspected intraabdominal bleeding d. Stabilize the patient. Then watch and wait. If the patient declines again in hematocrit levels then transfer to the Angio- suite

ChatGPT

In the scenario described, a 45-year-old female with blunt abdominal trauma, free abdominal fluid detected on Focused Assessment with Sonography for Trauma (FAST), and a falling haematocrit, the primary concern is internal bleeding and the need for prompt and effective management.

The most appropriate management option would be:

**a. Stabilize the patient. Then transfer to CT immediately. Depending on the findings consider Angio-suite or operating theater.**

Here's why:

1. **Stabilization First:** Initial stabilization is crucial in trauma patients. This involves ensuring adequate airway, breathing, and circulation (the ABCs of trauma care). Given the patient's falling haematocrit, it's imperative to manage potential shock, which may include fluid resuscitation and blood transfusions.
2. **Diagnostic Imaging (CT Scan):** After stabilization, the next step is usually diagnostic imaging, particularly a CT scan, to assess the extent and nature of the internal injuries. CT scans are more detailed than FAST and can help locate the source of bleeding and assess the severity of internal injuries.
3. **Decision Based on CT Findings:** Depending on the CT findings, the patient may then be transferred to an operating theater for surgery or the Angio-suite for angiographic embolization. The choice depends on the source and severity of the bleeding, the patient's overall stability, and other injuries.

Options b, c, and d are less appropriate because:

- **b (Immediate Surgery):** This might be necessary for cases of hemodynamic instability not responsive to initial resuscitation, but the scenario does not explicitly state that the patient is hemodynamically unstable.
- **c (Immediate Angio-suite):** While angiography and embolization are options for controlling hemorrhage, immediate transfer to the Angio-suite without a CT scan might bypass critical information needed for appropriate management.
- **d (Watch and Wait):** Given the evidence of internal bleeding (falling haematocrit and free fluid on FAST), a watchful waiting approach could be dangerous, as it might lead to a delay in necessary intervention.

Therefore, option a is the most comprehensive and appropriate choice, addressing both immediate stabilization needs and allowing for an informed decision regarding subsequent intervention based on detailed imaging findings.

Which imaging modality it’s the most appropriate with the highest sensitivity to detect the source of a suspected arterial bleeding in the trauma patient? Select one: a. Digital subtraction angiography b. MRA c. Triple phase CTA d. Ultrasound
