# Supplementary material for: GPT-4o’s competency in answering the simulated written European Board of Interventional Radiology exam compared to a medical student and experts in Germany and its ability to generate exam items on interventional radiology: a descriptive study
Source: J Educ Eval Health Prof. 2024 Aug 20;21:21. doi: 10.3352/jeehp.2024.21.21 (PMC11894030; doi:10.3352/jeehp.2024.21.21)
Supplement: Supplementary file 3 — Supplement 3. Fifty European Board of Interventional Radiology preparation items randomly selected from 370 items for 37 topics of the Cardiovascular and Interventional Radiology Society of Europe academy. [file jeehp-21-21-suppl3.docx]

**CIRSE PREP**

**1)**

An 83-year-old male patient with ischaemic cardiomyopathy (on treatment) and a heavy smoking history developed typical clinical and laboratory signs of acute mesenteric ischaemia.

CT angiogram demonstrated acute thrombosis of proximal portal vein and SMV, edema of mesenteric tissues and minimal thickening of the right colon wall.

Are there indications for transhepatic catheterisation and thrombolytic infusion?

Select one:

a. If thrombosis involves the portal vein, a transhepatic approach is not feasible. Only a transjugular route may be attempted. Systemic anticoagulation alone is not enough in cases of massive thrombosis like this one.

b. Guidelines stated that transhepatic or transjugular superior mesenteric vein catheterisation and thrombolytic infusion is never indicated in acute vein mesenteric ischaemia.

c. Venous mesenteric ischaemia may respond to systemic anticoagulation alone. Transhepatic or transjugular superior mesenteric vein catheterisation and thrombolytic infusion, depending on the severity of symptoms, condition, and response to systemic anticoagulation.

d. Venous mesenteric ischaemia may respond to systemic anticoagulation alone. But simultaneous transhepatic or transjugular superior mesenteric vein catheterisation and thrombolytic infusion may improve clinical success.

**2)**A 78-year-old female patient with hyperlipidaemia and history of dyspepsia and post-prandial abdominal discomfort experienced 3 months of post-prandial vomiting and a reduced appetite. Which one of the following findings does not represent an indication for mesenteric angioplasty and or stent placement in chronic mesenteric ischaemia?

Select one:
a. Arcuate ligament syndrome revealed by imaging

b. Involvement of at least two of the three visceral arteries

c. Heavily calcified stenosis/occlusion

d. Clinical triad of unintentional weight loss, postprandial abdominal pain and 2 diseased vessel

**3)**
Α 75-year-old female complains of bilateral intermittent claudication at 100 meters. She has a medical history of arterial hypertension for 20 years under medication, and diabetes mellitus for the past 15 years controlled with oral medications. ABI is 0.6 bilaterally. CT-angiography was performed that revealed bilateral occlusion of the common iliac artery. What is the TASC II classification?

Select one:
a. TASC A

b. TASC D

c. TASC C

d. TASC B

**4)**A 47-year-old female patient with a history of a non-resectable pancreatic head mass and jaundice was referred for percutaneous biliary drainage after a failed endoscopic procedure. After successful catheterisation of a dilated bile duct in the right hepatic lobe, cholangiography revealed an abrupt obstruction of the common bile duct proximal to the level of the papilla of Vater. Crossing of the ampulla was not feasible so an external biliary drain was placed with the tip positioned within the common bile duct proximal to the obstruction. The following day severe haemobilia was observed. The blood within the drainage bag was red. The patient was haemodynamically stable. A CT angiography was performed that did not show any signs of active extravasation. What treatment option would be best pursued next?

Select one:
a. Conservative treatment with FFP and blood products, repeat CT scan if haemobilia continues

b. Transfer the patient to an angiosuite and perform portal venography

c. Cap the drain for 2 days, and if bleeding continues after uncapping, perform a hepatic arteriogram

d. Transfer patient to the angiography suite and perform intra-arterial hepatic angiography

**5)**A 62-year-old female patient presented to the emergency department with painless obstructive jaundice and pruritus. Upon clinical examination she also mentioned weight loss of 10 kg within the previous month. The total bilirubin level was 290 μmol/l (normal range 1-17 μmol/l). An abdominal CT scan revealed the presence of a mass of 5 cm in maximum diameter at the level of the hilum, normal-sized liver lobes and patency of the left and right portal veins. Endoscopic retrograde cholangiopancreatography (ERCP) was attempted but this failed. A percutaneous biopsy was also performed that showed cholongiocarcinoma. It was non-resectable and the patient was referred for percutaneous biliary drainage and stenting. A right-sided cholangiogram revealed the presence of stricture at the level of the right hepatic duct-common hepatic duct without communication with the left hepatic duct. Crossing of the obstruction was managed with the use of a hydrophilic guidewire and an internal/external drainage catheter was placed. What treatment option would be best pursued next?

Select one:
a. Negotiate left hepatic duct from the right side

b. Puncture left side and drain left side as well

c. Apply dressing and transfer patient to the ward

d. Insertion of a metallic stent

**6)**

A 46-year-old diabetic patient is scheduled for endovascular treatment of a symptomatic carotid artery stenosis due to hypoechogenic vulnerable plaque. Which of the following statements concerning the plaque stability and carotid stenting is correct?

Select one:

a. Statin administration during the carotid artery stenting procedure and the use of stents with a small free cell area

b. Statin administration prior to carotid artery stenting and the use of stents with an open-cell design, because the stent needs to be flexible enough to accommodate to the tortuosity

c. Statin administration prior to carotid artery stenting and the use of stents with an open-cell design

d. Statin administration prior to carotid artery stenting and the use of stents with a small free cell area

**7)**

A 75-year-old patient with 80% left-sided internal carotid artery (ICA) stenosis and ipsilateral external carotid artery (ECA) occlusion is planned for a carotid artery stent (CAS). Stenosis or occlusion of the ECA can complicate sheath access because it cannot be used to anchor the exchange guidewire. Which of the following techniques is best for overcoming this issue?

Select one:

a. Telescoping technique

b. Direct carotid artery puncture

c. Radial or brachial approach

d. Subclavian “anchor” technique

**8)**

Which of the following statements regarding the position of the catheter tip of a CVA catheter is correct?

Select one:
a. Dialysis catheters should be placed in the proximal SVC near the brachiocephalic veins

b. One vertebral body height below the carina is a good position in most cases.

c. Groshong valves are associated with an increased risk of catheter thrombosis

d. Tunnelled catheters tend to move less with change of position compared to untunnelled catheters

**9)**

The rationale behind the efficacy of thermal ablation for treating liver tumours is the absolute sensitivity of any biological tissue to the heat. According to this concept, which is the range of temperature for achieving a “near instantaneous coagulative necrosis”?

Select one:
a. 60°C – 100°C

b. 46°C - 48°C

c. 50°C – 52°C

d. 110°C – 120°C

**10)**

Y90 radioembolisation (SIRT) is a well-tolerated intra-arterial therapy for metastatic liver disease. Among the following clinical issues, which one has to be considered an absolute contraindication?

Select one:

a. Total bilirubin level of 4.8 mg/dl

b. 4% lung shunting at MAA scintigraphy

c. Chronic renal failure

d. Bilobar liver disease

**11)**

A 65-year-old woman with breast cancer is referred for the curative treatment of a 30mm iliac metastasis. She is to undergo a cryoablation. In the largest studies focusing on cryoablation for local control for oligometastatic musculoskeletal disease, what is the size threshold which positively predicts favourable outcomes?

Select one:

a. 5mm

b. 25mm

c. 20mm

d. 30mm

**12)**

You have just performed a testicular varicocele embolisation in a young adult patient. When is the most appropriate timing for follow-up ultrasound?

Select one:

a. After one year if there are no symptoms

b. After 3 months

c. There is no need for an ultrasound if no symptoms

d. After 2 weeks

**13)**

A 14-year-old is referred to your clinic by their family doctor with Grade I testicular varicoceles. What is the most appropriate treatment plan?

Select one:

a. No treatment is necessary

b. Testicular varicocoele embolisation is recommended

c. Surgery is recommended

d. Refer to paediatric surgery for discussion and treatment after parental consent

**14)**

An 80-year-old male patient with toe gangrene is scheduled for a complex three-vessel infrapopliteal endovascular procedure. What is the optimal medication regime prior to such a complex infrapopliteal procedure?

Select one:

a. Single antiplatelet therapy with aspirin 75-100mg once daily is recommended.

b. Dual antiplatelet therapy (clopidogrel plus aspirin) should be prescribed at least three days before the procedure

c. Antiplatelet therapy should be discontinued to avoid bleeding events.

d. Single antiplatelet therapy with clopidogrel 75mg once daily is recommended.

**15)**

Which of the following is the most important risk factor for post-EVAR rupture?

Select one:

a. Type I endoleak

b. Age >75

c. Diameter of aneurysm sac > 65 mm

d. Type II endoleak

**16)**A 55-year-old female patient presents with ongoing disabling intermittent claudication <100m in spite of a 6-week supervised exercise programme. She underwent a successful percutaneous revascularisation of a non-calcified mid-superficial femoral artery 5-cm segment occlusion. What is the recommended endovascular treatment option in her case?

Select one:

a. Plain balloon angioplasty with bail-out stenting in case of suboptimal balloon angioplasty

b. Direct placement of a bare-metal stent

c. Primary placement of a paclitaxel-eluting stent

d. Paclitaxel-coated balloon angioplasty with bail-out stenting in case of suboptimal balloon angioplasty

**17)**

A 75-year-old patient underwent a biopsy of an abdominal mass.

A few months later, he reattended with a subcutaneous hard nodule. Ultrasound showed a hypoechoic subcutaneous nodule close to the entry point of the previous biopsy. Which initial pathology is most likely to cause this?

Select one:

a. A renal cell carcinoma

b. A renal lymphoma

c. A hepatocellular carcinoma

d. A liver metastasis

**18)**

A 75-year-old patient is scheduled for a biopsy of a liver mass in a cirrhotic liver. In the pre-procedural phase, how should the risk of procedural bleeding be assessed?

Select one:

a. A drug history of which anticoagulant/anti-platelet therapies are usually taken by the
patient.

b. INR, aPTT, platelet count and a drug history of which anticoagulant/antiplatelet therapies are usually taken by the patient.

c. INR and a drug history of which anticoagulant/anti-platelet therapies are usually taken by the patient.

d. INR and platelet count

**19)**

A 71-year old female underwent right hemi hepatectomy with Roux-en-Y hepatico-jejunostomy for an intrahepatic cholangiocarcinoma. Five days after surgery, she complained of abdominal pain. Vital signs were normal: blood pressure 150/85 mmHg, heart rate 70/min and temperature 37.4°C. Ultrasound of the abdomen demonstrated free fluid in the abdomen. Most fluid was located at the liver resection plane. MRI with a hepato-biliary specific contrast agent was performed. Images obtained 20 minutes after contrast injection demonstrated leakage of the contrast agent from the bilio-digestive anastomosis. What is the most appropriate management?

Select one:

a. Ultrasonographic puncture of the collection with sample collection for microbiological examination, followed by percutaneous drainage if the collection proves to be infected.

b. Endoscopic retrograde cholangiography with placement of a biliary stent

c. Drainage of para-hepatic fluid collection followed by percutaneous transhepatic biliary drainage if biliary leakage persists

d. Ultrasonographic puncture of the collection with sample collection for biochemical examination, followed by percutaneous drainage if the peritoneal fluid-to-serum bilirubin is >5.

**20)**

A 72 year old female presented to the emergency room with upper abdominal and fever. Her previous history included diabetes mellitus type II, hypertension and coronary artery disease. Blood tests showed an elevated white blood cell count and c-reactive protein. Ultrasound and CT demonstrated an 8cm abscess in the right liver lobe. The patient was transferred to the interventional radiology suite for percutaneous drainage. Prophylactic antibiotics were administrated and a 10F pigtail catheter was inserted under ultrasonographic guidance. Several minutes after placement of the catheter, the patient became hypotensive (90/60mmHg) and tachycardic (120/min). 500ml of fresh blood was seen in the drainage bag. What is the most appropriate next step?

Select one:

a. Fluid resuscitation and upsizing of the drainage catheter to 12F catheter

b. Immediate activation of the medical emergency team

c. Fluid resuscitation and urgent triple phase CT of the liver

d. Fluid resuscitation and hepatic angiography

**21)**

A 65-year-old woman has just had a foraminal steroid infiltration. She is taking oral metformin for her diabetes. What is the most appropriate advice regarding the effect of this treatment on her diabetes?

Select one:

a. She should double her daily dosage

b. She should start insulin treatment immediately

c. She should be informed to monitor her blood sugar levels, and contact her physician if they rise and be aware that she might need to increase her dosage.

d. She should consult with us in a couple of days about her sugar levels

**22)**

Which are the most important aspects to consider when choosing the appropriate ablation technique for a lung tumour?

Select one:

a. Diameter of the lesion, position (central or peripheral), proximity to mediastinum and large vessels, coagulation status, presence of

pace-maker.

b. All thermal ablation techniques have the same indications: the choice is only between a thermal or a non-thermal ablation (IRE).

c. The choice is made on the basis of the operator confidence.

d. The presence of pace-maker and the location of the lesion influence your choice

**23)**

Which is the most appropriate clinical decision-making in acute limb ischaemia (ALI)?

Select one:

a. Catheter-directed therapy should be initiated at all times

b. Viable limb – imaging – angioplasty

c. Threatened limb – heparin – imaging – catheter-directed therapy

d. Irreversible ischaemia – heparin – imaging – catheter-directed therapy

**24)**

A 40-year-old man with history of bronchiectasis is hospitalised and he is on intravenous antibiotics for fevers and purulent sputum production. On his second day in the hospital, the intern is called to his bedside because he has coughed up approximately 100 mls of dark blood with clots and he is expectorating more blood at the time of evaluation. On physical exam, he is afebrile with a pulse of 90 beats per minute, a blood pressure of 120/80, respiratory rate of 30 breaths per minute, and pulse oximetry of 94% on 2 litres of oxygen via nasal cannulae. He appears to be in mild respiratory distress. He has bilateral crackles on auscultation. CT angiography reveals ground glass opacities with diffuse bronchiectasis bilaterally. Bronchial artery angiography reveals bilateral tortuous bronchial arteries with pulmonary hypervascularity and blush. Embolisation is indicated. What is the best choice of embolic material in this case?

Select one:
a. Gelfoam

b. Polyvinyl alcohol (PVA) particles 350-500μm

c. Coils

d. Particles 100-300μm

**25)**Six weeks after surgical creation of a radiocephalic arteriovenous fistula (AVF), a 63-year-old patient underwent dialysis. Dialysis nurses reported difficulties during venous puncture, followed by very small flow ineffective dialysis. Duplex ultrasound showed: radial artery patent, regular anastomosis, narrow draining vein with two venous side branches out of the fistula, volumetric flow was <200 ml/min. What is the most appropriate treatment plan?

Select one:

a. Due to early fistula failure, surgical ligation of the existing AVF and creation of the new one in the more proximal aspect of the limb is the method of choice

b. Connecting venous side branches in an end to end manner with fistula reformation at the same site

c. Embolisation of the multiple venous side branches to redirect the flow to the main vein, together with a long-segment balloon angioplasty of the proximal venous aspect is recommended in this case of inadequate maturation

d. Multiple venous side branches embolisation in order to redirect the flow to the main vein, together with stent implantation in the proximal 5 cm of the vein is recommended in case of inadequate maturation

**26)**

A 66-year-old female patient presents with a complete occlusion of her upper arm brachiocephalic fistula. Palpation indicates occlusion of the vein from the anastomotic site. Which is the most appropriate management?

Select one:

a. It is recommended to start by a retrograde puncture and perform a thrombectomy of the proximal fistula segment close to the arterial anastomosis first

b. A single high cephalic vein puncture will give adequate access for fistula thrombectomy

c. Aneurysms of the upper cephalic vein contraindicate percutaneous thrombectomy as they are difficult to treat percutaneously

d. A double puncture in both retrograde and antegrade directions is recommended to cover the whole thrombosed segment

**27)**

A 56-year-old female has a single 2 cm osteoblastic metastasis in the body of her sternum. There is no soft tissue involvement or extension through the periostium. No other metastases are on PET-CT. The right heart is contacting with the sternal body. Which ablation method is appropriate in this setting?

Select one:

a. Radiofrequency ablation

b. Microwave ablation

c. External radiotherapy

d. Cryoablation

**28)**

Which of the following statements regarding the avascular plane of Brodel is correct?

Select one:
a. It is an avascular plane in the Gerota’s fascia.

b. It is the section of renal parenchyma between the anterior 2/3rd and posterior 1/3rd of the kidney on the cross-section that is relatively avascular.

c. If the puncture is performed in this plane the complication rate is 0%.

d. It is a small volume sited in the lower pole of the kidney with no vascularisation.

**29)**

What is the most serious complication of percutaneous nephrostomy (PCN) placement and why?

Select one:
a. Ureteral or renal pelvis injury.

b. Bleeding.

c. Septic shock.

d. Pleural complication.

**30)**

A severely osteoporotic patient is scheduled for an L1 vertebroplasty to treat a painful acute vertebral compression fracture (VCF). The patient is seen in consultation one week before the procedure and is advised about the risk of new VCFs in the adjacent vertebral levels. What is the level of this risk?

Select one:
a. Significantly Reduced

b. Significantly increasedSignificantly increased

c. Not significant

d. Significantly increased even if a very small amount of PMMA leaks in the T12-L1 inter-vertebral disc

**31)**

A 13-year-old girl is known to have a low-flow venous malformation affecting her right (dominant) forearm flexor compartment

presents with a painful, swollen and hot forearm for the past three days. What best describes her situation?

Select one:
a. She has developed an infection in the malformation which should be treated with antibiotics.

b. The malformation has become symptomatic, and as it is in her dominant arm, it should be treated with direct stick sclerotherapy.

c. She is experiencing an episode of acute thrombophlebitis and should be treated with a short course of anti- inflammatories.

d. She is experiencing growth of the venous malformation due to puberty. She should be monitored closely and only undergo treatment if progression is apparent.

**32)**

A 56-year-old man with a recently diagnosed symptomatic arteriovenous malformation type 3b (multiple shunts between arterioles and venules) on his neck is referred to you for therapy. Which of the following represents the preferred treatment option for this case?

Select one:
a. Percutaneous arterial approach, preferably transfemoral, and coil embolisation with dense coil-packing

b. Percutaneous arterial approach, preferably transfemoral, and embolisation with liquid embolic agents, such as EVOH

c. Direct puncture of the nidus under sonography and sclerotherapy with sodium tetradecyl sulphate (STS) or polidocanol

d. There is no indication for treating this kind of lesion. Clinical and radiological follow-up as well as changes in lifestyle (smoking cessation, exercises, optimising arterial blood pressure) should be performed

**33)**

A 39-year-old woman after vaginal delivery presents with loss of about 1000 ml of blood. She has no comorbidities or coagulopathy but conservative treatments have not stopped the bleeding. What is the most appropriate next action?

Select one:
a. Abdominal and pelvic CT angiogram to check for the source of bleeding

b. Embolisation of uterine arteries with resorbable material

c. Watch and wait unless the patient’s conditions deteriorate

d. Occlusion of uterine artery with coils

**34)**

A 32-year-old primigravida is experiencing significant blood loss following delivery of her baby at 41 weeks at a tertiary referral hospital. Conservative measures including transfusion (6 units in total), uterine massage and balloon tamponade of the uterus have all failed and she continues to haemorrhage. She is referred to interventional radiology for treatment. Which of the following is likely to be the best course of action?

Select one:
a. If no focal bleeding point is identified, carry out embolisation of bilateral internal iliac arteries using a non-permanent embolic agent such as Gelfoam.

b. Carry out an angiogram and only proceed to embolisation if a focal bleeding point is identified.

c. Insist on a CT angiogram before proceeding to the interventional radiology suite.

d. Given the extent of blood loss, it should be recommended that she proceeds directly to a hysterectomy to save her life.

**35)**

A 79-year-old man with long-term medical therapy of his lower urinary tract symptoms (**LUTS**) would like to know more about the course of symptom relief following **PAE**. Which statement related to follow-up after **PAE** is correct?

Select one:
a. The immediate symptom relief directly after **PAE** is attributable to nitric oxide (NO) release from the infarcted tissue

b. Lower urinary tract symptoms (**LUTS**) after prostate artery embolisation (**PAE**) improve constantly in the first 6 months, so follow-up imaging should only be performed after this time-period

c. Repeat-**PAE** of the already treated prostate is to be avoided, as no further benefit is to be expected

d. Prostatitis after **PAE** is a frequent complication

**36)**

Which of the below statements is the most appropriate relating to stochastic effects?

Select one:
a. The severity of the response is proportional to the dose.

b. They include skin lesions.

c. The probability of occurrence increases with increased dose absorption.

d. They have a threshold dose.

**37)**

An interventional radiologist would like to make sure to protect her eyes from radiation-induced cataract. What occupational dose limit for the lens of the eye should she avoid exceeding?

Select one:
a. 1 mSv/yr

b. 150 mSv/yr

c. 50 mSv/yr

d. 20 mSv/yr

**38)**

A 41-year-old man presented with a 4 cm mass in the lower pole of the left kidney, incidentally found on an abdominal ultrasound scan. The patient was referred for a staging CT scan that revealed the presence of a hypervascular mass of 4 cm, in the lower pole of the left kidney with tumour thrombus extending within the renal vein, without the presence of distant metastases. Percutaneous biopsy was performed which confirmed the presence of renal cell carcinoma, clear cell type. What is the incidence of this histologic type among renal cell carcinomas?

Select one:
a. 75-85%

b. 55-65%

c. 25-35%

d. 45-55%

**39)**

A 77-year-old male patient on Coumadin for DVT presents with an enlarging, hypervascular biopsy proven RCC of 5.5 cm in maximum diameter, in the mid pole of the right kidney, abutting the renal collecting system. CT scan depicted no metastases. MRI scan revealed significant stenosis of the left renal artery. His creatinine level is 133 μmol/L (normal range 50-120 μmol/L). Differential renal function revealed that the main function is from the right kidney. Patient first had left renal artery angioplasty and his creatinine level dropped to 110 μmol/L. Percutaneous cryoablation was decided. What is the most likely complication due to the proximity of the tumour to the collecting system?

Select one:
a. Renal artery injury

b. Arteriovenous fistula formation

c. Urinoma formation

d. Haematuria

**40)**

2 days post renal transplantation a 45-year-old patient develops high volume urine drainage from a peri-renal surgical drain. There is low volume drainage from the bladder catheter. Ultrasound reveals a large peri-transplant fluid collection (most likely a urinoma) and this is felt to be secondary to ureteric necrosis. What is the most appropriate treatment?

Select one:
a. Placement of a percutaneous urinoma drain

b. Surgical revision

c. Urinary decompression by nephrostomy

d. Urinary decompression by nephrostomy and internal double-J-stent

**41)**

A 63-year-old woman with atrial fibrillation suffered from onset of vertigo, nausea and drowsiness 6 hours ago. What are the main differences in assessment criteria when considering this patient with basilar artery occlusion as a candidate for endovascular therapy (in comparison to anterior circulation ischaemic stroke)?

Select one:

a. Mechanical thrombectomy is performed in everyone with basilar artery occlusion regardless of the time from onset of symptoms because of their very poor prognosis

b. The prognosis for these patients is different after intravenous and endovascular therapy.

c. ASPECTS, prognosis, evidence for endovascular therapy effectiveness versus intravenous thrombolysis

d. Patients with posterior circulation ischaemia are assessed according the same criteria as are used for anterior stroke; there are no differences

**42)**A 77-year-old male presents with superior vena cava compression syndrome (Kishi score 5). On contrast-enhanced CT, a large mediastinal and pleural tumour with SVC obstruction is present. Core needle biopsy proved this lesion as malignant mesothelioma. Based on imaging characteristics, it is staged as International Mesothelioma Interest Group Stage III. He also suffers thrombocytopenia with a platelet count of 90 x109/L. Laboratory testing also shows a moderately elevated C-reactive protein.

What type of treatment do you recommend to alleviate symptoms?

Select one:
a. Timely surgical resection

b. Urgent radiation therapy

c. SVC stenting

d. Urgent chemotherapy

**43)**

A 74-year-old male patient with recent onset chest and back pain of 4 days duration is referred by his general practitioner to the out-patient department. The patient has noticed minor occasional haemoptysis over the past 3 weeks. He has been a smoker for over 40 years and has chronic obstructive lung disease. The CTA shows a 6.8 cm diameter TAA of the distal thoracic aorta commencing 2.2 cm distal to the origin of the left subclavian artery and extending to 4.4 cm proximal to the celiac trunk. The diameter of the non-aneurysmal aorta proximal to the aneurysm is 34 mm and distal to the aneurysm 25 mm. In addition, an irregular central pulmonary nodule (1.9 cm) is seen in the right upper lobe. Which of the following would be the best possible treatment option?

Select one:
a. Bronchoscopy to rule out malignancy followed by elective TEVAR with 38 mm endografts proximally and distally.

b. Early TEVAR with 36 mm diameter endografts proximally and distally. Thereafter, elective bronchoscopy.

c. Order custom-made endograft with 38 mm proximal and 30 mm distal diameters. Perform bronchoscopy whilst awaiting endograft arrival.

d. Early TEVAR with a 30 mm diameter endograft distally followed by a 38 mm diameter endograft proximally. Thereafter, elective bronchoscopy.

**44)**

or which indication is Transjugular Intrahepatic Porto-systemic Shunt creation (TIPS) most frequently performed?

Select one:
a. Variceal bleeding refractory to other therapy

b. Liver cirrhosis without hepatic encephalopathy

c. Decompensated liver cirhosis

d. Refractory ascites in liver cirrhosis

**45)**

During a TIPS procedure, when puncturing the right portal vein branch from the right hepatic vein, in which direction should the TIPS needle be aimed?

Select one:
a. The middle hepatic vein should be catheterised since its course is in close anatomical proximity of the portal vein.

b. Posteriorly.

c. Anteriorly.

d. Strictly caudal

**46)**

A 37-year-old female with a history of multiple spontaneous abortions now presents with an acute deep-vein thrombosis (DVT). What is the most likely diagnosis?

Select one:
a. Autologous anti-heparin antibodies (AAHA)

b. Homozygous Factor V Leiden deficiency

c. Anti-phospho-lipid syndrome

d. Homozygous homocystinuria

**47)**

The most common type of endoleak following endovascular repair of the internal iliac artery aneurysm is:

Select one:
a. Type 1b

b. Type II

c. Type III

d. Type Ia

**48)**

A 47-year-old female has symptoms of frequent urination and pelvic pain. MR showed a 7.2 cm subserosal fibroid (in the right side of the uterus) and a 2.7 cm intramural fibroid. At angiography, what angle would you use first to visualise the origin of the left uterine artery?

Select one:
a. Ipsilateral 40°

b. RAO 40°, because the large fibroid is predominantly on the R side of the uterus

c. Contralateral 40°

d. AP

**49)**

A 65-year-old patient with intermittent claudication was successfully treated with angioplasty of the left superficial femoral artery via a 6 Fr antegrade access to the ipsilateral common femoral artery. The puncture site was successfully closed with a Starclose vascular closure device (VCD). Which of the following statements regarding the Starclose VCDs is correct?

Select one:
a. The left groin can be repunctured immediately.

b. Success rates after Starclose are higher than other VCDs.

c. The left groin cannot be repunctured immediately, because resorption of the nitinol clip takes approximately 6 weeks.

d. Due to the low profile of the applicator, widening of the subcutaneous access canal is not necessary.

**50)**

An 85-year-old male patient suffered blunt abdominal trauma. A contrast-enhanced CT scan shows a shattered spleen with multiple areas of active arterial bleeding. The patient is haemodynamically unstable with low-volume fluid support. What is the most appropriate management?

Select one:
a. Don’t embolize send patient for surgery

b. Proximal embolization using temporary gel foam embolization

c. Distal selective embolization of all appropriate branches

d. Proximal embolization of the splenic artery using coils or Amplatzer plugs

EBIR Level Set Items generated by GPT-4o

1)
Which of the following is not an indication for endovascular intervention in mesenteric ischemia?
a. Acute arterial embolism
b. Chronic mesenteric venous thrombosis
c. Atherosclerotic stenosis causing chronic ischemia
d. Non-occlusive mesenteric ischemia (NOMI) unresponsive to medical management

2)
What is the main treatment approach for mesenteric venous thrombosis?
a. Surgical intervention
b. Endovascular therapy
c. Conservative treatment
d. Immediate bowel resection

3)
In the context of TASC II D lesions in the aorto-iliac segment, which approach is generally recommended?
a. Primary angioplasty without stenting
b. Endovascular stent grafting as a first-line treatment
c. Surgical bypass as the preferred initial treatment
d. Sequential balloon angioplasty followed by stenting

4)
What is the primary advantage of self-expandable metal stents (SEMS) over plastic stents in the management of malignant biliary obstruction?
a. Lower cost
b. Easier to remove
c. Longer patency periods
d. Fewer complications

5)
When performing a percutaneous transhepatic biliary drainage, which of the following is the most common site for the initial puncture?
a. Left lateral duct
b. Right posterior duct
c. Right anterior duct
d. Common bile duct

6)
During carotid artery angioplasty, which type of stent is preferred for a tortuous internal carotid artery?
a. Closed-cell design stent
b. Open-cell design stent
c. Self-expanding nitinol stent
d. Balloon-expandable stent

7)
Which complication is most commonly associated with carotid artery angioplasty and stenting?
a. Hyperperfusion syndrome
b. Restenosis
c. Local hematoma at the puncture site
d. Cranial nerve injury

8)
In Y90 radioembolization for CRLM, what is the significance of a lung shunt fraction greater than 20% observed in pre-treatment planning?
a. It indicates a need for dose reduction.
b. It suggests the existence of hepato-pulmonary artery shunts.
c. It is a marker for increased risk of post-embolization syndrome.
d. It necessitates conversion to a different modality of treatment.

9)
In the setting of CRLM, what is the typical embolic agent used in transarterial chemoembolization (TACE) with drug-eluting beads loaded with Irinotecan (DEBIRI)?
a. Polyvinyl alcohol particles
b. Starch microbeads
c. Biocompatible microspheres
d. Lipiodol

10)
In the context of central venous access in patients with chronic renal failure, what is a major consideration when choosing the site for catheter placement?
a. Avoiding the subclavian vein
b. Preference for the femoral vein
c. Using the internal jugular vein
d. Prioritizing external jugular vein

11)
When considering cryoablation for the treatment of a vertebral metastasis, which anatomical consideration is most crucial to prevent complications?
a. Proximity to the spinal canal
b. Proximity to the vertebral artery
c. Position relative to the pedicles
d. Location within the vertebral body (anterior vs. posterior)

12)
In endovascular varicocele treatment, the "sclerotherapy-only" technique typically involves the use of which agent?
a. Ethanol
b. Cyanoacrylate glue
c. Sodium tetradecyl sulfate
d. Polidocanol

13)
During endovascular embolization of a varicocele, which anatomical variation most significantly increases the complexity of the procedure?
a. Retro-aortic left renal vein
b. Circumaortic left renal collar
c. Duplication of the inferior vena cava
d. Left-sided inferior vena cava

14)
For patients with critical limb ischemia undergoing infrapopliteal revascularization, which of the following factors most significantly impacts long-term limb salvage?
a. Post-procedural ankle-brachial index (ABI)
b. Type of anticoagulation used post-procedure
c. Extent of wound infection pre-procedure
d. The presence of a continuous single-vessel run-off to the foot

15)
When considering EVAR in patients with challenging neck anatomy, which graft feature is most beneficial for short, angulated necks?
a. Suprarenal fixation
b. Increased graft diameter
c. Branched stent grafts
d. Low-profile delivery systems

16)
What is the most common cause of early stent thrombosis in arterial interventions?
a. Inadequate antiplatelet therapy
b. Mechanical failure of the stent
c. Hypercoagulable state of the patient
d. Infection of the stent

17)
Which of the following factors most significantly influences the risk of needle tract seeding during an image-guided biopsy of a hepatocellular carcinoma?
a. Needle gauge size
b. Depth of the lesion within the liver
c. Type of imaging modality used for guidance
d. Duration of the needle within the lesion

18)
During an ultrasound-guided biopsy of a pancreatic mass, which of the following approaches minimizes the risk of fistula formation?
a. Transduodenal approach
b. Transgastric approach
c. Transhepatic approach
d. Transperitoneal approach

19)
When performing image-guided percutaneous drainage of a subphrenic abscess, which approach minimizes the risk of pleural transgression?
a. Subxiphoid approach
b. Transhepatic approach
c. Intercostal approach
d. Translumbar approach

20)
In image-guided drainage of a pancreatic pseudocyst, which factor regarding the drainage catheter is of most importance when the cyst content is viscous?
a. The tip-configuration of the catheter
b. The diameter of the catheter
c. If the catheter has a lumen for flushing
d. The coating of the catheter

21)
When conducting a CT-guided celiac plexus neurolysis, which anatomical landmark is essential for accurate needle placement?
a. Anterior to the aorta at the level of L1
b. Inferior to the superior mesenteric artery origin
c. Between the origins of the celiac trunk and the superior mesenteric artery
d. Lateral to the aorta at the level of the celiac trunk

22)
What is an important consideration when performing cryoablation for lung metastases?
a. Ensuring rapid thawing to prevent damage to surrounding tissues
b. The necessity of multiple freeze-thaw cycles to ensure complete ablation
c. The use of cryoablation is limited to central lesions only
d. Avoidance of cryoablation in patients with pre-existing pulmonary fibrosis

23)
What is the primary mechanism of action for catheter-directed thrombolysis in acute limb ischemia? a. Vasodilatation of affected vessels.
b. Mechanical removal of the thrombus.
c. Conversion of plasminogen to plasmin.
d. Platelet aggregation inhibition.

24)
Which embolic agent is preferred for permanent occlusion in the management of hemoptysis secondary to bronchiectasis?
a. Gelfoam
b. Particles
c. Coils
d. Glue

25)
In the case of an occluded arteriovenous fistula with no clear entry site for percutaneous access, what is the most appropriate initial approach?
a. Surgical exploration and thrombectomy
b. Ultrasound-guided puncture of the occluded segment
c. Systemic thrombolysis
d. Direct puncture of the arterial anastomosis

26)
Question 3: For a patient with a dialysis fistula and symptomatic ischemic steal syndrome, which surgical intervention is most appropriate?
a. Fistula ligation
b. Banding of the fistula
c. Distal revascularization with interval ligation (DRIL)
d. Proximalization of arterial inflow (PAI)

27)
In the context of osteolytic metastases, which of the following agents is most commonly used in percutaneous vertebroplasty to provide structural support?
a. Polymethylmethacrylate
b. Hydroxyapatite
c. Calcium phosphate cement
d. Iodine-containing acrylic cement

28)
In managing malignant ureteric obstruction, which of the following is NOT a typical consideration when choosing between a ureteral stent and a nephrostomy tube?
a. Patient's life expectancy
b. The presence of vesicoureteral reflux
c. The level of obstruction
d. Patient's preference and quality of life

29)
During percutaneous nephrolithotomy (PCNL) for a calyceal stone in a patient with a narrow infundibulum, which of the following is the most appropriate management strategy for a minor calyceal tear?
a. Surgical exploration
b. Placement of a double-J-catheter
c. Conservative management with a nephrostomy tube in place
d. Administration of a diuretic intraoperatively

30)
Which of the following is a relative contraindication for percutaneous vertebral augmentation?
a. Osteoporotic vertebral compression fracture
b. Vertebra plana
c. Acute traumatic vertebral fracture in a young adult
d. Vertebral hemangioma with aggressive features

31)
In the context of Klippel-Trenaunay Syndrome, which imaging feature is most indicative of a potentially high-risk venous malformation?
a. Presence of phleboliths on plain radiography
b. Enlarged deep venous channels on MRV
c. Hypertrophy of the affected limb
d. Skin discoloration overlying the malformation

32)
Which of the following molecular targeted therapies has shown efficacy in the treatment of complex lymphatic malformations unresponsive to conventional therapy?
a. Bevacizumab
b. Rapamycin
c. Trastuzumab
d. Vemurafenib

33)
In the setting of postpartum hemorrhage, embolization of the uterine artery should be avoided in which of the following conditions?
a. Known fibroids
b. Previous Cesarean section
c. Pre-existing pelvic inflammatory disease
d. Confirmed placenta increta

34)
When performing a pelvic angiogram for postpartum hemorrhage, visualization of which collateral pathway is crucial to prevent non-target embolization during uterine artery embolization?
a. Ovarian artery to uterine artery anastomosis
b. Internal pudendal artery to uterine artery anastomosis
c. Inferior mesenteric artery to internal iliac artery anastomosis
d. Superior gluteal artery to uterine artery anastomosis

35)
What is the most common variant of prostatic arterial supply in the context of prostate artery embolization?
a. The prostatic artery originating from the internal pudendal artery
b. The prostatic artery originating directly from the anterior division of the internal iliac artery
c. The prostatic artery originating from the obturator artery
d. The prostatic artery having a common origin with the superior vesical artery

36)
For dose optimization in interventional radiology, what is the impact of using a higher kVp and lower mAs setting?
a. Higher contrast resolution but increased patient dose
b. Lower patient dose but reduced image quality
c. Improved penetration with reduced skin dose
d. Decreased penetration with increased contrast dose

37)
How does doubling the distance between the source and the interventional radiologist affect the radiation exposure?
a. It reduces exposure by a factor of two
b. It reduces exposure by a factor of four
c. It reduces exposure linearly
d. It has no significant effect on exposure

38)
In the management of renal cancer, what is the primary indication for preoperative embolization?
a. Reduction of tumor size before cryoablation.
b. Minimization of blood loss in patients scheduled for nephrectomy.
c. Palliation of symptoms in inoperable cases.
d. Prevention of metastasis in high-grade tumors.

39)
Regarding renal angiomyolipomas, which statement accurately reflects the current understanding of their management in interventional radiology?
a. Prophylactic embolization is routinely recommended for lesions larger than 2 cm.
b. Angiomyolipomas larger than 4 cm should always be treated due to the high risk of rupture.
c. Aneurysms within angiomyolipomas pose the greatest risk of rupture and hemorrhage.
d. Embolization is contraindicated in angiomyolipomas with minimal fat content.

40)
In renal transplant recipients with refractory hypertension and suspected renal artery stenosis, which hemodynamic parameter measured during angiography is most predictive of a positive response to angioplasty?
a. Translesional systolic pressure gradient >10 mmHg
b. Translesional mean pressure gradient >15 mmHg
c. Renal-aortic ratio >0.90
d. Pulmonary capillary wedge pressure reduction post-angioplasty

41)
In stroke patients with known hereditary hemorrhagic telangiectasia (HHT), what factor most critically influences the approach to endovascular intervention?
a. Presence of pulmonary arteriovenous malformations (AVMs).
b. Severity of epistaxis.
c. Family history of brain AVMs.
d. History of previous gastrointestinal bleeding.

42)
The 'Kishi score' in the context of superior vena cava stenting for malignant obstruction is used to:
a. Assess the severity of SVC obstruction
b. Determine the risk of stent migration
c. Evaluate the likelihood of post-stenting restenosis
d. Quantify the extent of collateral venous circulation

43)
When considering spinal cord ischemia in the context of TEVAR, which collateral network is primarily responsible for the blood supply to the critical watershed area of the lower thoracic spinal cord?
a. Anterior spinal artery.
b. Artery of Adamkiewicz.
c. Internal iliac artery collaterals.
d. Vertebral artery pial network.

44)
During a TIPS procedure, coil embolization of the gastrorenal shunt is performed to reduce the risk of what specific complication?
a. Hepatic encephalopathy
b. Variceal rebleeding
c. Acute kidney injury
d. Stent migration

45)
In TIPS procedures, the use of direct intrahepatic portocaval shunts (DIPS) compared to conventional TIPS is indicated in which scenario?
a. Chronic portal vein thrombosis with cavernous transformation
b. Patients with Child-Pugh A cirrhosis
c. In the setting of acute variceal bleeding
d. When large spontaneous splenorenal shunts are present

46)
Regarding the use of ultrasound-accelerated thrombolysis in acute iliofemoral DVT, what is the primary benefit of ultrasound assistance in the context of thrombolytic therapy?
a. Increased penetration of thrombolytic agents into the thrombus
b. Direct mechanical breakup of the thrombus
c. Enhancement of fibrinolytic activity by altering fibrin structure
d. Reduction of the required dose of thrombolytic agent

47)
In the setting of a large isolated iliac artery aneurysm with a short (<10mm) infrarenal aortic neck, which of the following considerations is most critical for endovascular repair?
a. The potential need for a chimney graft for renal arteries
b. The risk of stentgraft migration into the aneurysm sac
c. The use of suprarenal fixation to secure the graft
d. The likelihood of needing adjunctive iliac branch devices

48)
In the context of uterine fibroid embolization (UFE), the "Ovarian Artery to Uterine Artery Collateral" (OAUAC) sign on angiography is indicative of:
a. Inadequate embolization of the uterine artery.
b. Potential risk for ovarian failure post-UFE.
c. Compensation due to chronic uterine artery occlusion.
d. Anatomical variation with negligible clinical significance.

49)
In the context of percutaneous transluminal angioplasty (PTA) of below-the-knee vessels, what is the optimal sheath-to-artery ratio to minimize the risk of access site complications in calcified vessels?
a. 1:1
b. Less than 1:1
c. Greater than 1:1 but less than 1.5:1
d. 1.5:1 or greater

50)
In managing a post-traumatic hepatic artery pseudoaneurysm with an aberrant right hepatic artery originating from the superior mesenteric artery, what is the critical factor to consider for successful embolization?
a. The presence of portal vein thrombosis
b. The size of the pseudoaneurysm
c. The flow dynamics in the collateral pathways
d. The diameter of the aberrant artery

Medical student Level Set Items generated by GPT-4o

1)
What often accompanies acute mesenteric artery thrombosis?
a. Vasculitis
b. Chronic atherosclerotic disease
c. Cardiac arrest
d. Hypercoagulability

2)
1What is the most common cause of acute mesenteric ischemia?
a. Mesenteric vein thrombosis
b. Non-occlusive mesenteric ischemia (NOMI)
c. Arterial embolism
d. Atherosclerotic disease

3)
What is the main advantage of using endovascular stenting over open surgical repair in aorto-iliac disease?
a. Lower risk of infection
b. Shorter hospital stay and quicker recovery
c. No need for general anesthesia
d. All of the above

4)
What is the most common complication associated with percutaneous biliary drainage?
a. Hemobilia
b. Cholangitis
c. Pancreatitis
d. Biliary leakage

5)
What is the primary indication for the placement of a biliary stent?
a. Chronic pancreatitis
b. Gallstone ileus
c. Obstructive jaundice due to malignancy
d. Acute cholecystitis

6)
Which imaging modality is essential for planning and guiding carotid artery angioplasty?
a. Ultrasound
b. MRI
c. X-ray
d. Digital subtraction angiography (DSA)

7)
Following carotid artery angioplasty, which of the following medications is typically prescribed to prevent stent thrombosis?
a. Antibiotics
b. Anticoagulants
c. Antihypertensives
d. Antiplatelet agents

8)
Percutaneous radiofrequency ablation (RFA) for colorectal liver metastases is typically indicated in which of the following scenarios?
a. Multiple bilobar liver metastases
b. Single or a few liver metastases, each smaller than 3 cm
c. Liver metastases with concurrent peritoneal carcinomatosis
d. Large liver metastases (>5 cm)

9)
What is the most common site of metastasis for colorectal cancer?
a. Lung
b. Brain
c. Liver
d. Bone

10)
1Which of the following is the preferred site for central venous catheter insertion due to its lower complication rates?
a. Femoral vein
b. Internal jugular vein
c. Subclavian vein
d. External jugular vein

11)
In treating oligometastatic musculoskeletal disease, which factor is crucial in determining the suitability of a patient for curative intent therapy?
a. Patient's age
b. Number of metastatic lesions
c. Type of primary cancer
d. All of the above

12)
What is the primary goal of endovascular treatment of varicoceles?
a. To reduce testicular size
b. To improve cosmetic appearance
c. To alleviate pain and improve fertility
d. To prevent testicular torsion

13)
Which vein is typically targeted in the endovascular treatment of left-sided varicoceles?
a. Left renal vein
b. Left internal iliac vein
c. Left gonadal vein
d. Left inferior epigastric vein

14) In below-the-knee endovascular therapy, what is the main purpose of using drug-coated balloons (DCBs)?
a. To mechanically remove plaques
b. To provide a permanent scaffold
c. To deliver a drug that inhibits restenosis
d. To temporarily expand the vessel

15)
What imaging modality is primarily used for planning an EVAR procedure?
a. Ultrasound
b. Plain abdominal X-rays
c. Computed Tomography (CT)
d. Magnetic Resonance Imaging (MRI)

16)
Which type of stent is commonly used in arteries prone to frequent movement and flexion?
a. Covered stents
b. Balloon-expandable stents
c. Self-expanding stents
d. Drug-eluting stents

17)
In a CT-guided lung biopsy, what is the most common complication?
a. Hemorrhage
b. Infection
c. Pneumothorax
d. Needle tract seeding

18)
Which imaging modality is preferred for guiding biopsies of superficial lymph nodes?
a. X-ray
b. Magnetic Resonance Imaging (MRI)
c. Ultrasound
d. Computed Tomography (CT)

19)
In image-guided drainage, what is the primary purpose of using a Seldinger technique?
a. To minimize the risk of infection
b. To ensure the precise placement of the drainage catheter
c. To provide a pathway for contrast media
d. To avoid the need for sedation

20)
What is the most likely complication following percutaneous drainage of a fluid collection using image guidance?
a. Anaphylaxis due to contrast media
b. Infection at the puncture site
c. Severe bleeding
d. Allergic reaction to anesthesia

21)
In image-guided pain therapy, what is the main purpose of using contrast agents during procedures like facet joint injections?
a. To alleviate pain
b. To confirm correct needle placement
c. To provide long-term therapeutic effects
d. To enhance the effect of the steroid medication

22)
What is a common complication associated with percutaneous ablation techniques for lung metastases?
a. Severe hemorrhage
b. Pneumothorax
c. Chronic cough
d. Fistula

23)
What is the initial clinical management for a patient presenting with acute limb ischemia?
a. Immediate amputation.
b. Systemic anticoagulation with unfractionated heparin.
c. Start oral anticoagulation with warfarin.
d. Rest and elevation of the limb.

24)
In the context of hemoptysis, which imaging modality is preferred for identifying the source of bleeding?
a. Standard chest X-ray
b. Bronchoscopy
c. CT angiography
d. MRI of the chest

25)
Which symptom is a common indication of a failing arteriovenous graft (AVG) for hemodialysis?
a. Increased venous pressure during dialysis
b. Persistent hypotension post-dialysis
c. Sudden weight gain between dialysis sessions
d. Elevated serum potassium levels

26)
What is the initial step in managing a stenosed arteriovenous fistula (AVF) used for hemodialysis?
a. Systemic anticoagulation therapy
b. Immediate surgical intervention
c. Percutaneous transluminal angioplasty (PTA)
d. Placement of a permanent central venous catheter

27)
What is the most common site for bone metastases?
a. Long bones
b. Skull
c. Spine
d. Pelvic bones

28)
In cases of pelvicalyceal and ureteric obstructions due to stones, which of the following is a standard initial management approach?
a. Immediate surgical intervention
b. Antibiotic therapy
c. Analgesics and hydration
d. Placement of a nephrostomy tube

29)
What is the most common complication following ureteral stent placement for obstruction?
a. Urosepsis
b. Stent migration
c. Urinary incontinence
d. Urinary tract infection

30)
What is the primary goal of percutaneous vertebral augmentation in osteoporotic fractures?
a. To restore the original shape of the vertebral body
b. To improve the patient's posture
c. To relieve pain and stabilize the fracture
d. To prevent future spinal cord injuries

31)
Question: What is the typical first-line treatment for a small, asymptomatic venous malformation?
a. Systemic corticosteroids
b. Sclerotherapy
c. Observation and monitoring
d. Surgical resection

32)
What is the most common type of vascular malformation?
a. Arteriovenous malformation (AVM)
b. Capillary malformation
c. Lymphatic malformation
d. Venous malformation

33)
What is the most common cause of primary postpartum hemorrhage?
a. Uterine atony
b. Retained placenta
c. Genital tract trauma
d. Coagulopathy

34)
In the context of postpartum hemorrhage, which medication is commonly used to contract the uterus?
a. Oxytocine
b. Ibuprofen
c. Amoxicillin
d. Metformin

35)
What is the primary indication for Prostate Artery Embolization?
a. Prostate cancer
b. Acute urinary retention
c. Benign prostatic hyperplasia
d. Chronic prostatitis

36)
The ALARA principle in radiation safety stands for:
a. As Long As Reasonably Achievable
b. As Low As Reasonably Achievable
c. Always Leave As Radioactive Areas
d. Advanced Level of Radiation Absorption

37)
In terms of radiation safety, what is the purpose of collimation in interventional radiology?
a. To focus the radiation beam on a specific area
b. To increase the intensity of the radiation
c. To warm up the equipment before use
d. To generate additional radiation for clearer images

38)
What is the most common presenting symptom of renal cell carcinoma?
a. Weight loss
b. Hematuria
c. Hypertension
d. Flank pain

39)
Which of the following is a known risk factor for the development of renal cell carcinoma?
a. High calcium intake
b. Chronic hepatitis C infection
c. Smoking
d. High Ibuprofene consumption

40)
What is the primary treatment for lymphocele causing obstruction in a transplant kidney?
a. Systemic antibiotics
b. Surgical drainage
c. High fluid intake
d. Corticosteroid therapy

41)
A patient with an acute stroke has a National Institutes of Health Stroke Scale (NIHSS) score of 20. How would you classify the severity of this stroke?
a. Minor.
b. Moderate.
c. Moderate to severe.
d. Severe.

42)
Which symptom is most commonly associated with the need for superior vena cava stenting in malignant obstruction?
a. Lower extremity edema
b. Ascites
c. Facial swelling and upper extremity edema
d. Abdominal pain

43)
Which imaging modality is essential for planning and guiding TEVAR procedures?
a. X-ray.
b. Ultrasound.
c. Computed Tomography Angiography (CTA).
d. Magnetic Resonance Imaging (MRI).

44)
What is the primary indication for performing a TIPS procedure?
a. Portal vein thrombosis
b. Hepatocellular carcinoma
c. Refractory variceal bleeding
d. Cholestatic jaundice

45)
Which parameter is crucial in assessing the immediate success of TIPS procedure?
a. Serum albumin level
b. Portosystemic gradient reduction
c. White blood cell count
d. Liver enzyme levels

46)
Which imaging modality is most commonly used to diagnose deep vein thrombosis?
a. X-ray
b. Venography
c. Doppler ultrasound
d. MRI

47)
A patient with an isolated iliac artery aneurysm presents with chronic back pain. The most likely cause of this symptom is:
a. Compression of the lumbar nerve roots by the aneurysm
b. Rupture of the aneurysm
c. Chronic inflammation around the aneurysm
d. Ischemia of the spinal cord

48)
Which imaging modality is most commonly used to assess the uterine anatomy and fibroid burden before uterine fibroid embolization?
a. X-ray.
b. Ultrasound.
c. Magnetic Resonance Imaging (MRI).
d. Computed Tomography (CT) scan.

49)
Which of the following is a benefit of using the radial artery for vascular access over the femoral artery?
a. Larger vessel diameter for easier access
b. Lower risk of major bleeding complications
c. More direct path to the heart
d. No risk of radial artery occlusion

50)
What is the initial imaging modality of choice for evaluating a patient with suspected vascular injury following a blunt trauma?
a. Plain radiography
b. Doppler ultrasound
c. Computed Tomography Angiography (CTA)
d. Magnetic Resonance Angiography (MRA)
